# Characterization of PC12 Cell Subclones with Different Sensitivities to Programmed Thermal Stimulation

**DOI:** 10.3390/ijms21218356

**Published:** 2020-11-07

**Authors:** Tada-aki Kudo, Kanako Tominami, Satoshi Izumi, Yohei Hayashi, Takuya Noguchi, Atsushi Matsuzawa, Guang Hong, Junichi Nakai

**Affiliations:** 1 Division of Oral Physiology, Tohoku University Graduate School of Dentistry, Sendai 980-8575, Japan; tominami@tohoku.ac.jp (K.T.); satoshi.izumi.b8@tohoku.ac.jp (S.I.); junichi.nakai.a5@tohoku.ac.jp (J.N.); 2 Cell Resource Center for Biomedical Research, Institute of Development, Aging and Cancer, Tohoku University, Sendai 980-8575, Japan; yohei.hayashi.e2@tohoku.ac.jp; 3 Graduate School of Life Sciences, Tohoku University, Sendai 980-8577, Japan; 4 Laboratory of Health Chemistry, Graduate School of Pharmaceutical Sciences, Tohoku University, Sendai 980-8578, Japan; takuya.noguchi.a7@tohoku.ac.jp (T.N.); atsushi.matsuzawa.c6@tohoku.ac.jp (A.M.); 5 Division for Globalization Initiative, Liaison Center for Innovative Dentistry, Tohoku University Graduate School of Dentistry, Sendai 980-8575, Japan; hong.guang.d6@tohoku.ac.jp

**Keywords:** bone morphogenetic protein (BMP), nerve growth factor (NGF), neuritogenesis, PC12 cells, thermal stimulation

## Abstract

Neuritogenesis is the process underling nervous system regeneration; however, optimal extracellular signals that can promote neuronal regenerative activities require further investigation. Previously, we developed a novel method for inducing neuronal differentiation in rat PC12 cells using temperature-controlled repeated thermal stimulation (TRTS) with a heating plate. Based on neurogenic sensitivity to TRTS, PC12 cells were classified as either hyper- or hyposensitive. In this study, we aimed to investigate the mechanism of hyposensitivity by establishing two PC12-derived subclones according to TRTS sensitivity during differentiation: PC12-P1F1, a hypersensitive subclone, and PC12-P1D10, a hyposensitive subclone. To characterize these subclones, cell size and neuritogenesis were evaluated in subclones treated with nerve growth factor (NGF), bone morphogenetic protein (BMP), or various TRTS. No significant differences in cell size were observed among the parental cells and subclones. BMP4- or TRTS-induced neuritogenesis was increased in PC12-P1F1 cells compared to that in the parental cells, while no neuritogenesis was observed in PC12-P1D10 cells. In contrast, NGF-induced neuritogenesis was observed in all three cell lines. Furthermore, a BMP inhibitor, LDN-193189, considerably inhibited TRTS-induced neuritogenesis. These results suggest that the BMP pathway might be required for TRTS-induced neuritogenesis, demonstrating the useful aspects of these novel subclones for TRTS research.

## 1. Introduction

Neuritogenesis is an important event in nervous system development and regeneration [1,2]. With the correct set of biological and physical stimuli, repair and/or regeneration of damaged neuronal tissue can occur [3,4]. Therefore, it is necessary to decipher optimal extracellular signals to promote neuronal regenerative activities, particularly those that induce cellular neurogenesis [5,6].

The PC12 cell line is a widely-used polyclonal cell line derived from rat adrenal pheochromocytoma cells and is an excellent model for the neuronal differentiation of mammalian cells [7,8,9,10,11]. It also has the potential to develop synapses with primary cultured neurons isolated from rat cerebral cortex [12]. PC12 cells represent a suitable model to study the autocrine/paracrine effects of endogenous nerve growth factor (NGF) and bone morphogenetic protein (BMP) signaling, as PC12 cells express receptors for both NGF and BMP [7,13]. The PC12 cells differentiate into neuron-like cells when exposed to neurotrophic factors, including NGF and BMPs) [14,15,16,17]. NGF binds to its membrane receptor, tropomyosin-related kinase A (TrkA), and BMPs such as BMP2 and BMP4, and associates with membrane heteromeric receptor complexes (BMP type I and II receptors). NGF and BMP activate their respective intracellular downstream pathways, inducing the neuronal differentiation of PC12 cells [18,19,20,21]. However, the rapid diffusion and short half-life of these humoral factors may limit their efficiency in vivo [4,22].

Studies have shown that thermal stimulation, as an alternative to neurotrophic factor treatments, has the potential to induce neuronal differentiation of mammalian cells [6,23]. For example, we previously reported the biological effects of a temperature-controlled repeated thermal stimulation (TRTS), a programmed thermal stimulation, on PC12 cells to induce neuritogenesis [6]. While the mode of action of TRTS at the cellular and molecular levels remains mostly unclear, we noticed that PC12 cells could be classified, based on their sensitivity to TRTS-induced neuritogenesis, into at least two types: hyper- or hyposensitive.

Because of the possible therapeutic applications of thermal stimulation, the purpose of this study was to investigate the mechanism underlying the differential sensitivity of PC12 cells to TRTS. Initially, we established two types of PC12-derived subclones according to TRTS sensitivity during neuronal differentiation: a TRTS-hypersensitive subclone (PC12-P1F1) and a TRTS-hyposensitive subclone (PC12-P1D10). To characterize these two models, which are novel and are expectedly suitable for studying TRTS, cell morphology and neuritogenesis were evaluated in subcloned cell lines treated with neurite inducers (NGF and BMP) or various types of TRTS. We also examined the possible underlying mechanism of TRTS action in PC12 cells.

## 2. Results and Discussion

**Figure 1:**
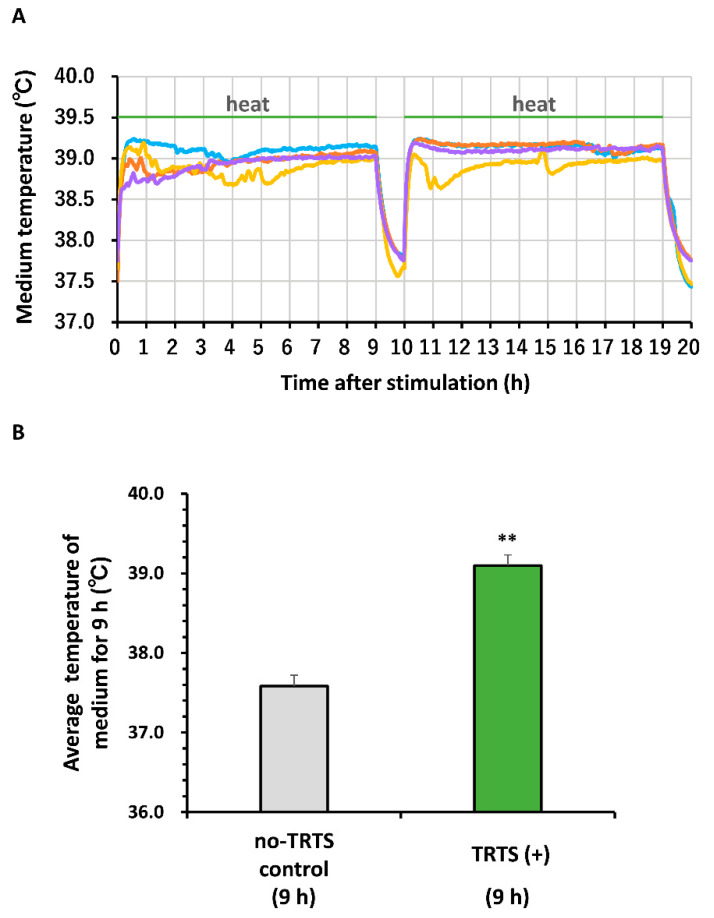
Temperature changes in the culture medium on a heating plate during temperature-controlled repeated thermal stimulation (TRTS). (**A, B**) One day before thermal evaluation, cell-free culture medium was transferred into a 24-well culture plate. The temperature of the medium was recorded every 60 s with a thermocouple data logger. (**A**) Temperatures of the culture medium during TRTS (for a total of 18 h) were recorded every 60 s. The data represent the overlayed temperature changes of four independent replicates. There was a 1 h break between the 9 h thermal stimulations. (**B**) Average medium temperatures with or without continuous heating for 9 h. The data represent the means ± standard deviation of the recorded medium temperatures for a typical sample during measurements in the presence or absence of TRTS. ** *p* < 0.01 vs. no-TRTS control.

To assess the thermal stability of TRTS, we measured the temperature of the culture medium with and without TRTS on the heating plate in the absence of cells to confirm the effect of TRTS on medium temperature. As shown in Figure 1A,B, upon TRTS exposure, the culture medium temperature rapidly increased and was maintained at an almost constant average level (near 39°C, range: 38.6–39.2°C) throughout the 9 h thermal stimulation period. Following thermal stimulation, the medium temperature gradually decreased throughout the interval period and returned to near the equilibrium temperature every time. On the other hand, in the absence of TRTS, the average culture medium temperature for the same 9 h period was maintained at an almost constant level (37.6°C) in the CO_2_ incubator throughout the entire measurement period (Figure 1B). These results confirm that the medium temperature was strictly and reproducibly controlled within a certain range during TRTS, as reported previously [6].

To establish PC12 subclones that were hypersensitive or hyposensitive to TRTS, single-cell cloning was performed using the limiting dilution method, obtaining 34 subclones. Through neuritogenesis assay, PC12-P1F1 and PC12-P1D10, subclones that were hypersensitive and hyposensitive to TRTS, respectively, were selected from the isolated subclones and were stored for subsequent analysis (for details, see Materials and Methods).

**Figure 2:**
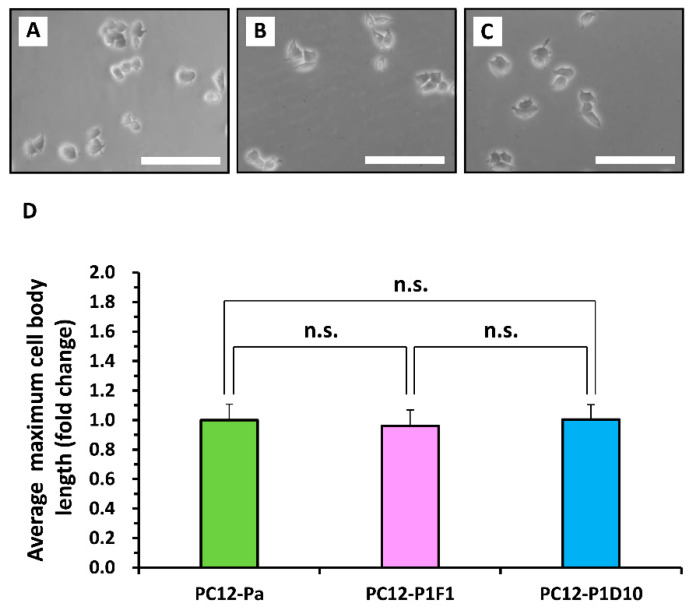
Comparison of cell morphology among the PC12, PC12-P1F1, and PC12-P1D10 cell lines. One day before microscopic observation, the parental PC12 cell line and its two subclones (PC12-P1F1 and PC12-P1D10) were seeded into culture medium on 24-well culture plates. (**A**–**C**) Representative phase-contrast micrographs of cultured cells from each cell line. Scale bars, 50 μm. Similar results were obtained in three independent experiments. (**D**) Average values of the maximum cell body length (*n* = 27) in each cell line calculated using data acquired from the phase-contrast micrographs. Results are presented as fold change relative to PC12-Pa. The data represent the means ± standard deviation of three replicates. PC12-Pa, parental PC12 cells; n.s., not significant.

Next, to compare morphologies between the parental PC12 cell line and its two subclones (PC12-P1F1 and PC12-P1D10) in the absence of TRTS, the three cell lines were seeded separately at the same time into growth medium on 24-well culture plates. Phase-contrast micrographs (Figure 2A–C) after 1 day of culture showed that all cell lines exhibited normal morphology with round and polygonal shapes, as described previously [8], and no apparent morphological difference was detected among these cell lines. Furthermore, no statistically significant difference was observed in the average value of the maximum cell body length obtained from the phase-contrast micrographs (Figure 2D).

**Figure 3:**
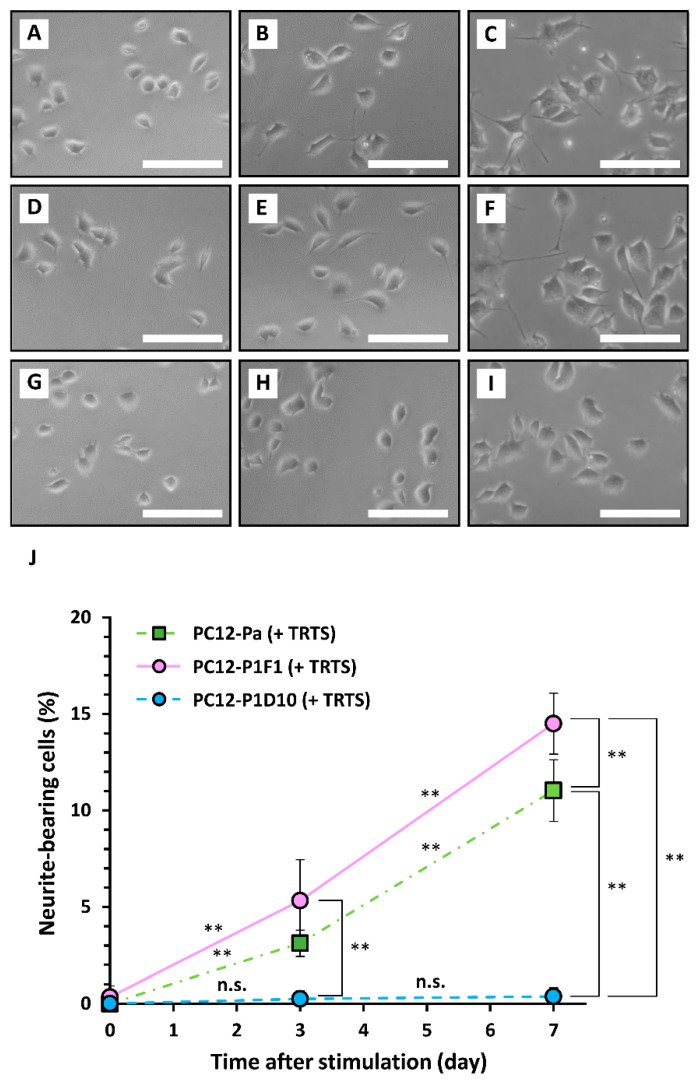
Time-course of temperature-controlled repeated thermal stimulation (TRTS)-induced neuritogenesis in PC12, PC12-P1F1, and PC12-P1D10 cells. Parental PC12, PC12-P1F1, and PC12-P1D10 cells were exposed to TRTS for an 18 h period each day for 7 days, and the extent of neuritogenesis was evaluated. Phase-contrast images of parental PC12 cells on day 0 prior to TRTS (**A**), and on days 3 (**B**) and 7 (**C**) after TRTS. Phase-contrast images of PC12-P1F1 cells on day 0 prior to TRTS (**D**), and on days 3 (**E**) and 7 (**F**) after TRTS. Phase-contrast images of PC12-P1D10 cells on day 0 prior to TRTS (**G**), and on days 3 (**H**) and 7 (**I**) after TRTS. Scale bars, 50 μm. Similar results were obtained in three independent experiments. (**J**) Parental PC12, PC12-P1F1, and PC12-P1D10 cells were exposed to TRTS for 18 h/day for 7 days, and the percentages of neurite-bearing cells on days 0, 3, and 7 were determined. The data represent the means ± standard deviation of three replicates. PC12-Pa, parental PC12 cells; n.s., not significant. ** *p* < 0.01.

**Figure 4:**
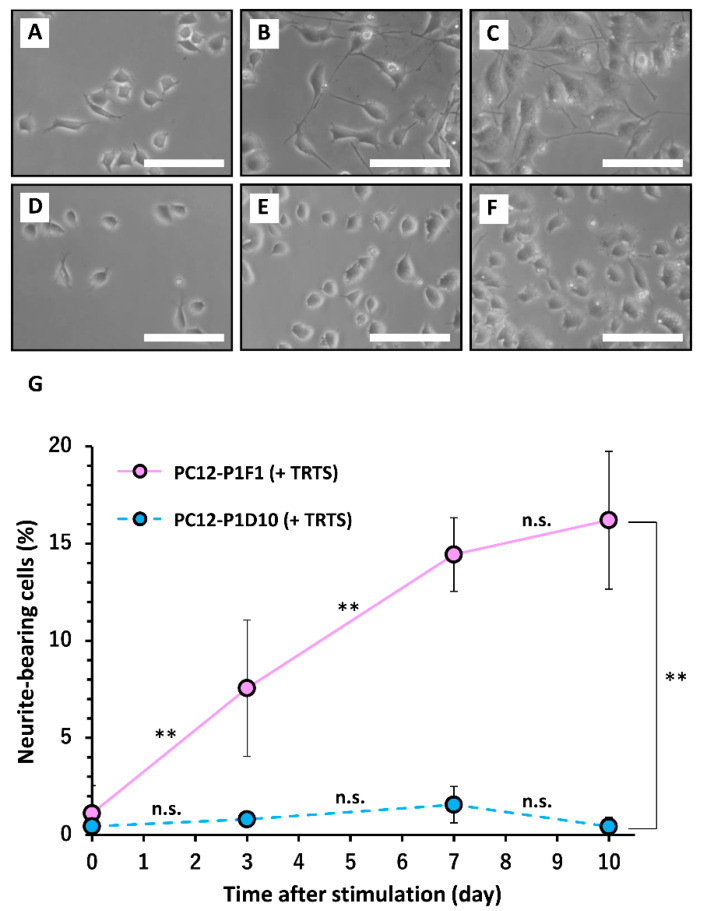
Time-course of temperature-controlled repeated thermal stimulation (TRTS)-induced neuritogenesis in the PC12 subclones. PC12-P1F1 and PC12-P1D10 cells were exposed to TRTS for an 18 h period each day for 10 days, and the extent of neuritogenesis was evaluated. Phase-contrast images of parental PC12-P1F1 cells on day 0 prior to TRTS (**A**) and on days 7 (**B**) and 10 (**C**) after TRTS. Phase-contrast images of PC12-P1D10 cells on day 0 prior to TRTS (**D**), and on days 7 (**E**) and 10 (**F**) after TRTS. Scale bars, 50 μm. Similar results were obtained in three independent experiments. (**G**) PC12-P1F1 and PC12-P1D10 cells were exposed to TRTS for 18 h/day for 10 days, and the percentages of neurite-bearing cells on days 0, 3, 7, and 10 were determined. The data represent the means ± standard deviation of three replicates. n.s., not significant. ** *p* < 0.01.

To compare the three cell line sensitivities to TRTS, each was exposed to TRTS for 7 days, and the extent of neuritogenesis was evaluated. As shown in Figure 3, prior to TRTS (day 0), the cells were relatively round and small with few visible neurites. TRTS-mediated neuritogenesis occurred gradually in a time-dependent manner in parental PC12 cells in the absence of other neuritogenesis inducers. TRTS-induced neuritogenesis was increased in PC12-P1F1 cells, compared to parental PC12 cells, while minimal neuritogenesis was observed in PC12-P1D10 cells on day 7 of the neuritogenesis assay (Figure 3). To investigate the possibility that additional TRTS exposure might promote belated neuritogenesis of PC12-P1D10 cells, we assessed the extent of neuritogenesis on day 10 in PC12-P1F1 and PC12-P1D10 cells. While neuritogenesis was increased to 16.2 ± 3.5% (*n* = 3) on day 10 in PC12-P1F1 cells, there was no significant neuritogenesis (0.4 ± 0.5% on day 10, *n* = 3) in PC12-P1D10 cells on the same day (Figure 4).

**Figure 5:**
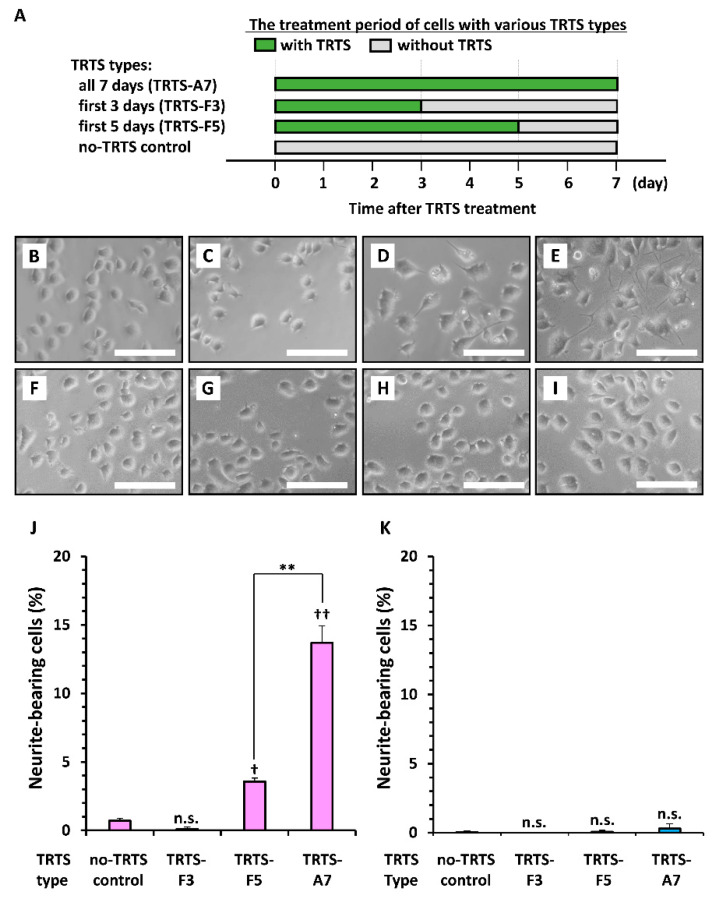
Effects of various types of temperature-controlled repeated thermal stimulation (TRTS) on neuritogenesis in PC12-P1F1 and PC12-P1D10 cells. PC12-P1F1 and PC12-P1D10 cells were exposed to TRTS for 18 h/day for the indicated days, and the extent of neuritogenesis was evaluated on day 7. (**A**) Schematic representation of the cell treatment periods with various TRTS types. The two PC12 subclones were exposed to TRTS for the following periods: all 7 days (TRTS-A7), first 3 days (TRTS-F3), first 5 days (TRTS-F5), or 0 days (as a no-TRTS control). Phase-contrast images of PC12-P1F1 cells on day 7 left untreated as a no-TRTS control (**B**) and on day 7 after TRTS-F3 (**C**), TRTS-F5 (**D**), or TRTS-A7 (**E**). Phase-contrast images of PC12-P1D10 cells on day 7 left untreated as a no-TRTS control (**F**) and on day 7 after TRTS-F3 (**G**), TRTS-F5 (**H**), or TRTS-A7 (**I**). Scale bars, 50 μm. Similar results were obtained in three independent experiments. PC12-P1F1 (**J**) and PC12-P1D10 (**K**) cells were exposed to TRTS-A7, TRTS-F3, or TRTS-F5, or left untreated as a no-TRTS control, and the percentages of neurite-bearing cells on day 7 were determined. The data represent the means ± standard deviation of three replicates. n.s., not significant. ** *p* < 0.01; ^†^, ^††^ *p* < 0.05, 0.01 vs. no-TRTS control, respectively.

We next assessed the possibility that varying the time-course of TRTS might change the extent of neuritogenesis in the subclones. We scored neuritogenesis in the two PC12 subclones exposed to TRTS for 18 h/day over the following periods: all 7 days, first 3 days, first 5 days, or 0 days as a no-TRTS control (Figure 5A). As shown in Figure 5B–K, TRTS-mediated neuritogenesis depended on the length of TRTS exposure in PC12-P1F1 cells but was absent in the presence of each tested TRTS in PC12-P1D10 cells on day 7. Overall, the results shown in Figure 3, Figure 4 and Figure 5 suggest that an increased daily load of TRTS is essential for more efficient TRTS-dependent neuronal differentiation. However, PC12-P1D10 cells completely lost the ability to elongate neurites in a TRTS-dependent manner.

**Figure 6:**
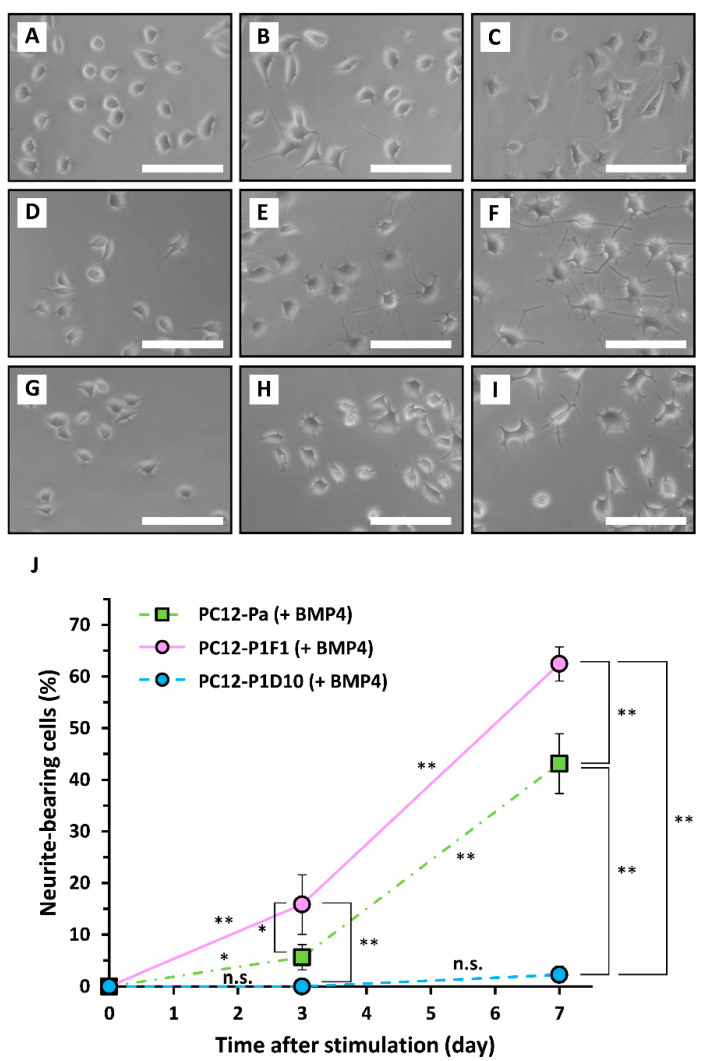
Time-course of bone morphogenetic protein (BMP)-induced neuritogenesis in parental PC12, PC12-P1F1, and PC12-P1D10 cells. Parental PC12, PC12-P1F1, and PC12-P1D10 cells were exposed to 40 ng/mL BMP4 for 7 days, and the extent of neuritogenesis was evaluated. Phase-contrast images of parental PC12 cells on day 0 prior to BMP4 exposure (**A**), and on days 3 (**B**) and 7 (**C**) after BMP4 exposure. Phase-contrast images of PC12-P1F1 cells on day 0 prior to exposure to BMP4 (**D**), and on days 3 (**E**) and 7 (**F**) after BMP4 exposure. Phase-contrast images of PC12-P1D10 cells on day 0 prior to exposure to BMP4 (**G**), and on days 3 (**H**) and 7 (**I**) after BMP4 exposure. Scale bars, 50 μm. Similar results were obtained in three independent experiments. (**J**) Parental PC12, PC12-P1F1, and PC12-P1D10 cells were exposed to 40 ng/mL BMP4 for 7 days and the percentages of neurite-bearing cells on days 0, 3, and 7 were determined. The data represent the means ± standard deviation of three replicates. PC12-Pa, parental PC12; n.s., not significant. *, ** *p* < 0.05, 0.01, respectively.

**Figure 7:**
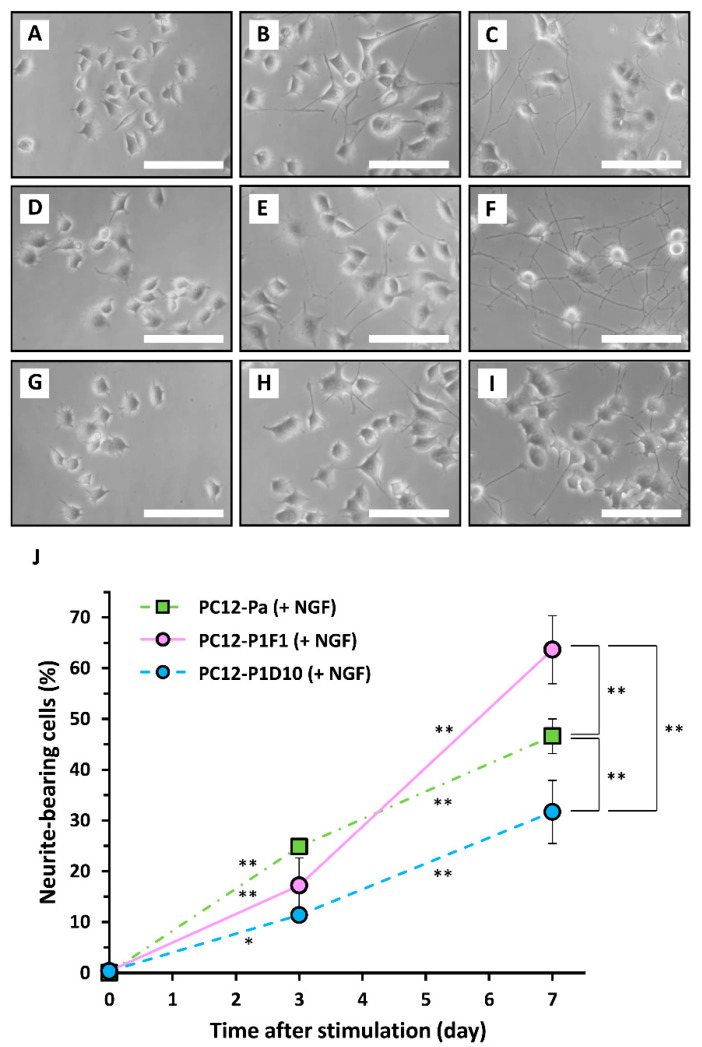
Time-course of nerve growth factor (NGF)-induced neuritogenesis in parental PC12, PC12-P1F1, and PC12-P1D10 cells. Parental PC12, PC12-P1F1, and PC12-P1D10 cells were exposed to 50 ng/mL NGF for 7 days, and the extent of neuritogenesis was evaluated. Phase-contrast images of parental PC12 cells on day 0 prior to NGF exposure (**A**), and on days 3 (**B**) and 7 (**C**) after exposure to NGF. Phase-contrast images of PC12-P1F1 cells on day 0 prior to exposure to NGF (**D**), and on days 3 (**E**) and 7 (**F**) after exposure to NGF. Phase-contrast images of PC12-P1D10 cells on day 0 prior to exposure to NGF (**G**), and on days 3 (**H**) and 7 (**I**) after NGF exposure. Scale bars, 50 μm. Similar results were obtained in three independent experiments. (**J**) Parental PC12, PC12-P1F1, and PC12-P1D10 cells were exposed to 50 ng/mL NGF for 7 days and the percentages of neurite-bearing cells on days 0, 3, and 7 were determined. The data represent the means ± standard deviation of three replicates. PC12-Pa, parental PC12. *, ** *p* < 0.05, 0.01, respectively.

To compare the sensitivities of parental PC12, PC12-P1F1, and PC12-P1D10 cells to neurotrophic factors, the cells were exposed to 40 ng/mL BMP4 or 50 ng/mL NGF for 7 days as positive controls, and the extent of neuritogenesis was evaluated. Treatment with BMP4 induced neuritogenesis gradually by day 7, not only in parental PC12 cells (43.1 ± 5.8% on day 7, *n* = 3) as observed previously [24] but also in PC12-P1F1 cells (62.5 ± 3.3% on day 7, *n* = 3) (Figure 6). In contrast, no significant enhancement of neuritogenesis was observed by day 7 in PC12-P1D10 cells exposed to BMP4 (2.3 ± 1.5% on day 7, *n* = 3) (Figure 6). This finding was similar to the lack of effect of TRTS on PC12-P1D10 cells (Figure 3). NGF-mediated neuritogenesis occurred gradually in a time-dependent manner in parental PC12 cells by day 7 (46.6 ± 3.4% on day 7, *n* = 3) (Figure 7). Moreover, NGF-mediated neuritogenesis was significantly higher in PC12-P1F1 cells (63.6 ± 6.7% on day 7, *n* = 3) and lower in PC12-P1D10 cells than in parental PC12 cells.

The precise reason for the partial inhibition of neuritogenesis in NGF-treated PC12-P1D10 cells compared to that in parental PC12 cells might involve an unknown PC12-P1D10-specific impairment of endogenous NGF-mediated signaling pathway(s) in differentiating PC12 cells, in addition to the observed inability of PC12-P1D10 cells to elongate neurites in response to BMP (Figure 6). However, we cannot exclude the possibility that other unknown mechanism(s) underlying the observed inability of PC12-P1D10 cells to elongate neurites upon BMP4 treatment might be involved in the observed partial inhibition of neuritogenesis in NGF-treated PC12-P1D10 cells. This is because the BMP signaling pathway might crosstalk with the NGF signaling pathway and enhance NGF responses during neuronal differentiation of PC12 cells [18,25,26]; however, the crosstalk mechanism of the two signaling pathways is not fully understood.

In this context, it should be noted that our previous study showed that the inhibition of TrkA (a membrane receptor of NGF) with a small-molecule inhibitor, GW441756, failed to inhibit TRTS (18 h/day)-mediated neuritogenesis in parental PC12 cells, whereas the inhibition of extracellular signal-regulated kinase activity with a small-molecule inhibitor, U0126, suppressed TRTS-induced neuritogenesis [6]. Thus, results imply that NGF-mediated TrkA might not be an essential signaling molecule of TRTS and is not directly involved in TRTS-mediated neuritogenesis. However, future analysis of TRTS-mediated intracellular signaling might be helpful for further understanding of the observed inhibition of neuritogenesis in NGF- or BMP-treated PC12-P1D10 cells.

**Figure 8:**
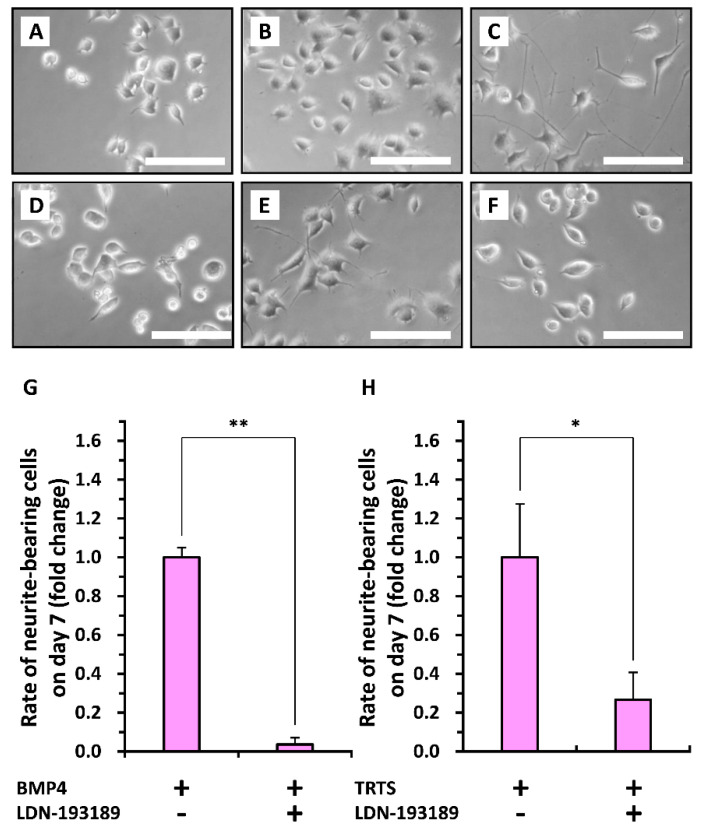
Suppression of temperature-controlled repeated thermal stimulation (TRTS)-induced neuritogenesis in PC12-P1F1 cells by a bone morphogenetic protein (BMP) signaling inhibitor. (**A**–**F**) PC12-P1F1 cells were exposed to TRTS (18 h/day) or left untreated as a control for 7 days in the presence or absence of 0.5 mM LDN-193189. Representative phase-contrast images of PC12-P1F1 cells on day 0 prior to TRTS exposure (**A**) and on day 7 after culture with no stimulation (**B**), or stimulation with 40 ng/mL BMP4 (**C**), 40 ng/mL BMP4 plus 0.5 mM LDN-193189 (**D**), TRTS (18 h/day) (**E**), or TRTS plus 0.5 mM LDN-193189 (**F**). Scale bars, 50 μm. Similar results were obtained in three independent experiments. (**G**–**H**) PC12-P1F1 cells were stimulated with 40 ng/mL BMP (**G**) or exposed to TRTS (18 h/day) (**H**) for 7 days, and the rate of neurite-bearing cells on day 7 was determined. Results are presented as fold change relative to BMP4- or TRTS-treated cells in the absence of 0.5 mM LDN-193189. The data represent the means ± standard deviation of three replicates. *, ** *p* < 0.05, 0.01, respectively.

Finally, to assess the role of BMP signaling in TRTS-dependent neuritogenesis, the extent of neuritogenesis was evaluated in PC12-P1F1 cells exposed to 40 ng/mL BMP4 (as a control) or TRTS (18 h/day) for 7 days in the presence or absence of 0.5 mM LDN-193189, a BMP signaling inhibitor that blocks the kinase activity of BMP type I receptors [27]. LDN-193189 treatment considerably inhibited both BMP- and TRTS-mediated neuritogenesis (Figure 8). These results suggest that the inhibition of BMP-mediated signaling at the membrane receptor level is sufficient to block TRTS-dependent neuritogenesis in PC12 cells, and TRTS-hyposensitive PC12-P1D10 cells are a subclone that has lost the ability to elongate neurites in a BMP4-dependent manner. 

Taken together, the results of the present study clearly show biological differences in the neurogenic potentials of the newly-generated PC12 subclones under the various investigated experimental conditions, principally using neurotrophic factors, and neuritogenesis assays. Although we did not evaluate the biochemical difference in the expression levels of GAP-43 [28], a representative biochemical marker for neuritogenesis, between parental PC12 cells and the subclones, our findings suggest that the BMP signaling pathway plays an essential role in TRTS-dependent neuronal differentiation of PC12 cells. The results obtained using the two subclones also indicate the existence of a cell type- or cell content-dependent mechanism that is involved in TRTS sensitivity for neuritogenesis, supporting the idea that parental PC12 cell line might be a polyclonal cell line with diverse sensitivities to TRTS. However, it remains largely unclear how TRTS-induced intracellular signaling is linked to the BMP signaling pathway and subsequent neuritogenesis in PC12 cells.

**Figure 9:**
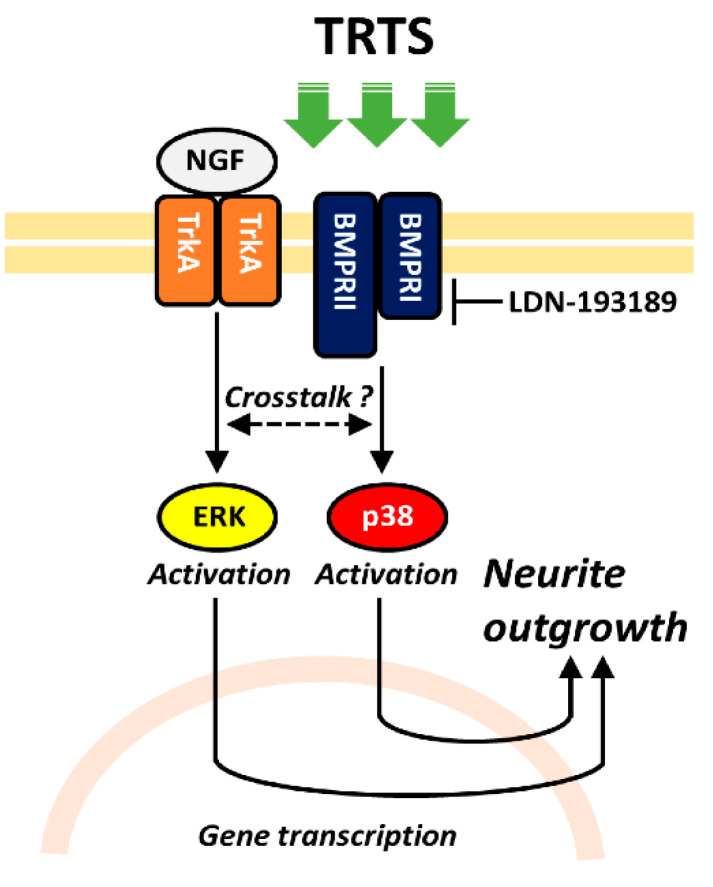
Proposed schema of the mechanisms by which LDN-193189 suppresses temperature-controlled repeated thermal stimulation (TRTS)-induced PC12 cell neuritogenesis. Hypothetically, treating PC12 cells with TRTS activates BMP receptor-mediated p38 MAPK signaling, which is essential for promoting the neuronal differentiation of PC12 cells. Thus, the inhibition of BMP type I receptor kinase activity with LDN-193189, a BMP signaling inhibitor, inhibits TRTS-mediated neuritogenesis in PC12 cells. This hypothesis also proposes an unknown crosstalk mechanism between the signaling pathways underlying BMP receptor- and NGF-mediated neuritogenesis. BMP, bone morphogenetic protein; BMPRI, BMP type I receptor; BMPRII, BMP type II receptor; ERK, extracellular signal-regulated kinase; NGF, nerve growth factor; TrkA, tropomyosin receptor kinase A.

Our previous study showed that the p38 mitogen-activated protein kinase (MAPK) inhibitor SB203580 was useful for inhibiting TRTS-induced neuritogenesis in PC12 cells [6]. Together with the current findings, these results raise a possibility that treating PC12 cells with TRTS might activate BMP receptor-mediated p38 MAPK signaling (Figure 9), which is known to be required for and can promote the neuronal differentiation of PC12 cells [19,29]. In this context, TRTS might first promote BMP autocrine/paracrine actions through a yet unrevealed mechanism that might involve the activation of BMP receptors in PC12 cells. However, this hypothesis was not verified in the present study. Besides, we cannot rule out the possibility that TRTS may directly affect the conformation of the BMP receptors, thus, rendering BMP receptors more sensitive to BMPs. Nevertheless, other yet unidentified mechanisms might be at play, and further studies are warranted to fully evaluate these hypotheses.

## 3. Materials and Methods

### 3.1. Cells and Reagents

Parental PC12 cells were provided by RIKEN BRC (Tsukuba, Japan). Recombinant human BMP4 (PeproTech, Rocky Hill, NJ, USA) and recombinant human β-NGF (PeproTech) were dissolved in LF6 buffer solution (5 mM glutamic acid, 5 mM NaCl, 2.5% glycine, 0.5% sucrose, 0.01% Tween 80, pH 4.5). LDN-193189, a selective inhibitor of BMP type I receptors [17,27], was obtained from Cayman Chemical (Ann Arbor, MI, USA) and dissolved in dimethyl sulfoxide (Fujifilm Wako Pure Chemical, Tokyo, Japan). Penicillin–streptomycin solution was from Sigma-Aldrich (St. Louis, MO, USA). Glutamic acid, NaCl, glycine and sucrose were from Fujifilm Wako Pure Chemical. Tween 80 was obtained from MP biomedicals (Solon, OH, USA).

### 3.2. Cell Culture and the Induction of Neurite Outgrowth

Parental PC12 cells and the variants were grown in Dulbecco’s modified Eagle’s medium (DMEM; Fujifilm Wako Pure Chemical) supplemented with 10% of a serum alternative (FetalClone III, GE Healthcare, Arlington Heights, IL, USA) and penicillin/streptomycin at 37 °C under 5% CO_2_ conditions. For the neuritogenesis assay, cells were seeded in the medium at a density of 1×10^4^ cells/well in collagen type IV-coated 24-well plates (Corning, Corning, NY, USA) and allowed to grow for 24 h. Subsequently, the cells were serum-starved in DMEM supplemented with 1% FetalClone III and penicillin/streptomycin and set on heating plates (Thermoplate II or III, Tokai Hit, Fujinomiya, Japan) to maintain the surface temperature during TRTS. For TRTS, cultures were heated to about 39 °C, a temperature optimized for neuronal differentiation [6], for a total of 18 h/day for various periods. For heat stimulation periods lasting longer than 9 h, a 1 h break was programmed to avoid long-term continuous stimulation using a PT70DW digital timer (REVEX, Kawaguchi, Japan), which can digitally control the on/off power period of the connected devices. In our previous study, a 3 h long heat stimulation was repeated 6 times to carry out a total 18 h long heat stimulation per day for TRTS [6]. However, in the present study, the process for TRTS was partially modified, and a 9 h long heat stimulation was repeated 2 times to achieve the same period of heat stimulation per day. Alternatively, cells were treated with 50 ng/mL NGF or 40 ng/mL BMP4 as positive controls for neuritogenesis [21,30]. The neuritogenesis assay was performed as described previously [6,17]. In brief, neuritogenesis was examined using a phase-contrast microscope (Leica DMIL LED microscope, Leica Microsystems, Wetzlar, Germany). Three images per well were acquired with a MC120 HD digital camera (Leica Microsystems) connected to the Leica Application Suite image capture software version 4.5 (Leica Microsystems). Cells bearing projections 1.5 times longer than the maximum cell body length were considered positive for neuritogenesis. If a cell had multiple projections or a projection had branches, the longest neurite length per cell was used for evaluation. At least 300 cells were evaluated per well. Each data point corresponded to the counts acquired from three independent wells.

### 3.3. Generation and Selection of PC12 Subclones

For single-cell cloning using the limiting dilution method, parental PC12 cells were grown in a 10-cm culture dish (Iwaki Glass, Chiba, Japan). After dissociation and counting the cell population in the logarithmic growth phase, an appropriate dilution of the cell suspension was made using a hemocytometer (Erma, Tokyo, Japan). Then, aliquots of the cell suspension that included just 1 cell per 0.2 mL medium were collected individually using a micropipette and placed separately in all wells of three 96-well plates (TrueLine, Nippon Genetics, Tokyo, Japan). After incubation, 34 successfully isolated subclones were propagated via passage into 24-well plates (TPP, Trasadingen, Switzerland). With the neuritogenesis assay described above, the TRTS-hypersensitive subclone, PC12-P1F1 (one of the most hypersensitive subclones to TRTS), and the TRTS-hyposensitive subclone, PC12-P1D10 (one of the most hyposensitive subclones to TRTS), were selected from the acquired subclones according to the percentage of positive cells for neuritogenesis after TRTS exposure (on day 7). The two selected subclones were stored in liquid nitrogen before further analysis.

### 3.4. Thermal Evaluation of the Medium

The temperature of the medium during heat stimulation was measured every 60 s as described previously [6]. We used a miniature thermocouple temperature sensor associated with the heating plate (Thermoplate II or III) adjusted to warm the medium to about 39 °C from the preset temperature of 37°C in a humidified incubator (APC-30D; Astec, Fukuoka, Japan) under 5% CO_2_ conditions. The temperature in the medium during TRTS was recorded using a TC-08 thermocouple data logger (Pico Technology, Cambridge, UK) and PicoLog 6 data logging software (Pico Technology).

### 3.5. Statistical Analyses

Data are presented as means ± standard deviation. Statistical analysis was performed with the statistical package JSTAT for Windows, version 6.8 (Sato, Japan). Significant differences between groups were identified by a one- or two-way analysis of variance followed by Tukey’s test or Student’s t-test, as appropriate. *p*-values < 0.05 were considered statistically significant.

## 4. Conclusions

In conclusion, we successfully established two new subclones of the PC12 cell line, PC12-P1F1, and PC12-P1D10, which are hypersensitive and hyposensitive for neuritogenesis to TRTS, respectively. We observed that the two subclones have typical morphologies (round and polygonal shapes with standard cell size) in undifferentiated states compared to that of the parental PC12 cell line and revealed their different neurogenic potentials by characterizing them in terms of their sensitivity for neuritogenesis to TRTS, as well as to other neurotrophic factors, namely NGF and BMP4. Specifically, BMP4- or TRTS-induced neuritogenesis was increased in PC12-P1F1 cells compared to parental PC12 cells, while no significant neuritogenesis was observed in PC12-P1D10 cells. In contrast, NGF-induced neuritogenesis was significantly induced in all three cell lines. Moreover, a selective inhibitor of BMP type I receptors, LDN-193189, substantially inhibited neuritogenesis induced by TRTS. Thus, our data raised the possibility that subjecting PC12 cells to TRTS can induce neuritogenesis, with the participation of the endogenous BMP signaling pathway, albeit via an unknown mechanism. These findings demonstrate the useful aspects of these novel PC12 subclones for analyzing TRTS-mediated signaling pathways for neuronal differentiation. Further research into the mechanisms underlying the action of TRTS and identifying the critical target signaling molecule(s) that lead to neuritogenesis in these cell lines will promote future applications of TRTS in studies of neuronal differentiation regulation.
